# Neurorepair and Regeneration of the Brain: A Decade of Bioscaffolds and Engineered Microtissue

**DOI:** 10.3389/fcell.2021.649891

**Published:** 2021-04-07

**Authors:** Laura N. Zamproni, Mayara T. V. V. Mundim, Marimelia A. Porcionatto

**Affiliations:** Molecular Neurobiology Laboratory, Department of Biochemistry, Escola Paulista de Medicina, Universidade Federal de São Paulo, São Paulo, Brazil

**Keywords:** bioscaffolds, biomaterials, brain repair, tissue engineering, stem cells

## Abstract

Repairing the human brain remains a challenge, despite the advances in the knowledge of inflammatory response to injuries and the discovery of adult neurogenesis. After brain injury, the hostile microenvironment and the lack of structural support for neural cell repopulation, anchoring, and synapse formation reduce successful repair chances. In the past decade, we witnessed the rise of studies regarding bioscaffolds’ use as support for neuro repair. A variety of natural and synthetic materials is available and have been used to replace damaged tissue. Bioscaffolds can assume different shapes and may or may not carry a diversity of content, such as stem cells, growth factors, exosomes, and si/miRNA that promote specific therapeutic effects and stimulate brain repair. The use of these external bioscaffolds and the creation of cell platforms provide the basis for tissue engineering. More recently, researchers were able to engineer brain organoids, neural networks, and even 3D printed neural tissue. The challenge in neural tissue engineering remains in the fabrication of scaffolds with precisely controlled topography and biochemical cues capable of directing and controlling neuronal cell fate. The purpose of this review is to highlight the existing research in the growing field of bioscaffolds’ development and neural tissue engineering. Moreover, this review also draws attention to emerging possibilities and prospects in this field.

## Graphical Abstract

Bioscaffolds potential applications in tissue engineering. Bioscaffolds can be used to grow stem cells and target their differentiation *in vitro* **(upper, left)** or be used as stem cell delivery route in a brain injury **(upper, right)**. Bioscaffolds can also contain si/miRNAs that will modify locally neural cells gene expression **(lower, left)** or contain exosomes/growth factors for paracrine signaling such as stimulating neurogenesis and increase neural stem migration to injury area **(lower, right)**. This cover has been designed using resources created by Vitaly Gorbachev from Flaticon.com.

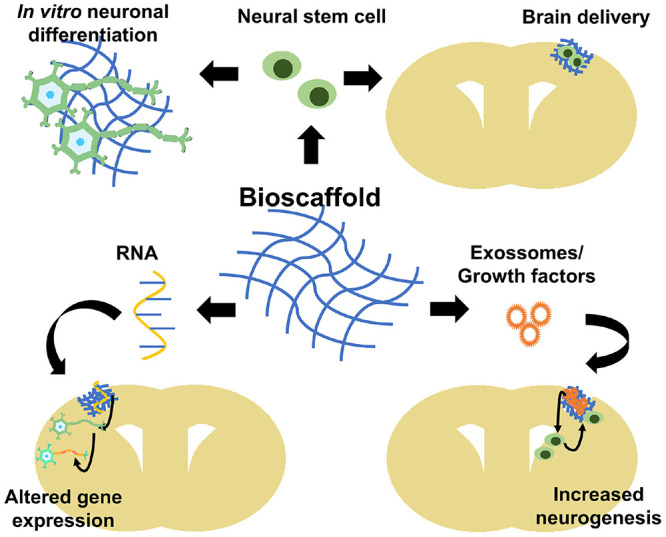

## Brain Injuries

Brain injuries are a significant cause of mortality and morbidity across the world. Injuries are divided into two types: (i) traumatic brain injury (TBI), caused by an external force to the head, such as a bump, blow, or penetrating object, and (ii) injury associated with a neurologic illness or condition, such as stroke, brain cancer, and other neurogenerative diseases (Stephenson et al., 2018; Cabrera, 2021). In 2016, neurological disorders were the world’s leading cause of disability-adjusted life-years, defined as the sum of years of life lost and years lived with disability, afflicting 276 million people and the second leading death cause, killing 90 million people (GBD 2016 Neurology Collaborators, 2019). The outcome of brain injuries is cell death, with high chances of functional and cognitive limitations, such as movement deficits, mood disorders, headaches, disturbances of memory, emotion, and behavior, and increased risk of development of neurodegenerative diseases (Riggio, 2011; Sulhan et al., 2020). Brain injuries reduce the quality of life of the injured person and their families, besides its high cost to healthcare systems (Humphreys et al., 2013).

## Cellular and Molecular Responses to Brain Injuries

### Inflammatory Response

Inflammation is a complex biological process in the body in response to cell and tissue damage (Chen et al., 2017). The definition of neuroinflammation is an inflammatory process within the brain or spinal cord (DiSabato et al., 2016; Wang Y. et al., 2020). Neuroinflammation is a common feature in many neurological diseases such as brain trauma, stroke, multiple sclerosis (MS), Alzheimer’s disease (AD), and Parkinson’s disease (PD) (Stephenson et al., 2018). Neuroinflammation will vary in type and range depending on the context, duration, and course of the primary insult. Inflammation can be transient and self-limited, facilitating tissue repair or persistent and dysregulated, leading to a chronic inflammatory state, resulting in tissue degeneration (Tansey et al., 2007).

There are several possible mechanisms of inflaming. Here, we provide a general overview of the process. The inflammatory processes may be initiated by the endogenous host-derived cell debris [damage-associated molecular patterns (DAMPs)] originated from acute cell death or that accumulate with age due to increased production or impaired elimination (Sochocka et al., 2017). DAMPs bind on pattern recognition receptors (PRRs), leading to cellular activation, that triggers inflammatory response. The PRRs comprise a family of membrane-bound toll-like receptors (TLRs), C-type lectin receptors (CLRs), cytoplasmic receptors, RIG-like receptors (RLRs), and NOD-like receptors expressed mainly on resident microglia (Dokalis and Prinz, 2019). Resident microglia are central players in this process because of their active role in immune surveillance. Microglia remove cell debris and become activated, releasing inflammatory proteins, like Interleukin 1 beta (IL1β), Interleukin 6 (IL 6), tumor necrosis factor alpha (TNF α), chemokines (such as C-C motif ligand 2 or CCL2 and C-X-C motif ligand 1 or CXCL1), reactive oxygen species (ROS) proteases and prostaglandins (Petrovic-Djergovic et al., 2016; Szalay et al., 2016; Mundim et al., 2019). Astrocytes become reactive, a process characterized by changes like hypertrophy and increased glial acid fibrillary protein (GFAP) expression (Wang et al., 2018). Reactive astrocytes proliferate and migrate through the injury site. Astrocytes secrete matrix metalloproteinases (MMPs) that degrade extracellular matrix (ECM) and facilitate their migration, but also degrade the basal lamina and promote blood–brain barrier (BBB) breakdown (Abdul-Muneer et al., 2016). With the disruption of the BBB, circulating neutrophils, monocytes, T cells, and dendritic cells invade the brain parenchyma and potentiates inflammation, creating a positive loop (Takeshita and Ransohoff, 2012).

**FIGURE 1:**
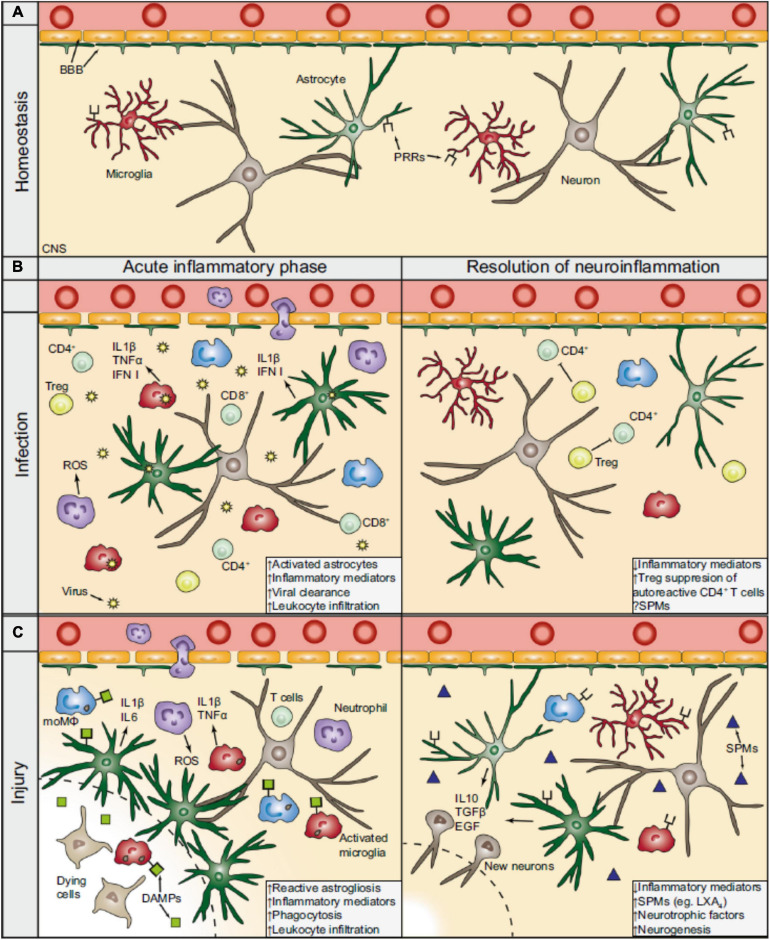
The inflammatory sequel during CNS viral infection and injury. **(A)** During homeostasis, the BBB separates the periphery from the CNS by tightly regulating the entrance of circulating molecules and nutrients. Astrocytes are part of the BBB and functionally support neurons, while microglia constantly survey the CNS parenchyma for potential factors that could compromise its integrity. **(B)** During viral infection, intracellular PRRs (not shown) recognize virus-derived material and activate resident CNS cells to produce cytokines (e.g., interferons), which recruit peripheral immune cells. CD4+ and CD8+ T cells (lime) play a prominent role for the effective clearance of the virus. During the resolution phase and after the clearance of the virus, Tregs (yellow) action is important in silencing autoreactive CD4+ T cells and promotes resolution. The contribution of SPMs following viral CNS infections remains largely unexplored. **(C)** In a generalized model of CNS injury, DAMPs are released from dying and stressed cells which bind to PRRs thereby activating the resident CNS cells. The BBB is consequently compromised, hypertrophic astrocytes surround the lesion core (dashed line), and microglia become activated. In turn, glial cells can release pro-inflammatory mediators (e.g., IL 1β), which attract peripheral leukocytes and augment the inflammatory response. During the resolution of the neuroinflammation following CNS injury, anti-inflammatory cytokines (e.g., IL 10) and trophic factors (e.g., EGF) are released, promoting neuronal tissue regeneration. SPMs, such as LXA4, that are produced at the injured parenchyma facilitate, as well, in the resolution phase. BBB, blood-brain barrier; CNS, central nervous system: PRRs, pattern recognition receptors; moMΦ, monocyte-derived macrophages (blue); ROS, reactive oxygen species; Treg, regulatory T cells; IFN, interferon; SPMs, specialized pro-resolving mediators; DAMPs, damage-associated molecular patterns; IL, interleukin; EGF, endothelial growth factors; LXA4, lipoxin A4. Extracted from Dokalis and Prinz (2019). Republished with Springer Nature permission.

The inflammatory response stop mechanism is called “inflammation resolution.” Inflammation resolution naturally occurs after acute or chronic inflammation and relies on the synthesis of specialized pro-resolving lipid mediators (SPM) by endothelial cells, macrophages, and neutrophils (Shang et al., 2019). SPM are a class of cell signaling molecules (Carracedo et al., 2019) that includes resolvins, protectins, maresins, and lipoxins (Qu et al., 2015). They result from the metabolism of polyunsaturated fatty acids released from omega-3-rich membranes by lipoxygenase, cyclooxygenase, or cytochrome P450 monooxygenase enzyme. During neuroinflammation resolution, anti-inflammatory cytokines as Interleukin 10 (IL 10) and trophic factors are released, promoting tissue regeneration (Figure 1; Dokalis and Prinz, 2019). However, if the inflammatory process remains unresolved, it can lead to chronic central nervous system (CNS) inflammation and neurodegeneration.

### Extracellular Matrix (ECM) Remodeling and Glial Scar

After an injury, the presence of ROS, free radicals, and pro-inflammatory cytokines makes the perilesional area a hostile environment for cell survival (Sulhan et al., 2020). Reactive astrocytes migrate to the injury site, where they secrete inflammatory factors and MMPs that remodel the ECM, creating a barrier between the injured and the healthy tissue (Jang et al., 2020). The glial scar consists predominately of reactive astrocytes, microglia/macrophages, and ECM molecules, mainly chondroitin sulfate proteoglycans (CSPG) (Rolls et al., 2009). The glial scar contains the spread of neurotoxic molecules and prevents the expansion of neuronal damage and degeneration. Thus, the glial scar is essential for preventing extra cell degeneration in injury’s acute phase (Rolls et al., 2009).

Astrocytes are well known for providing neuron trophic support. In the injury site, astrocytes maintain that function, producing and secreting several metabolites, including glucose, nutrients, and growth factors such as insulin-like growth factors (IGFs), nerve growth factors (NGF), brain-derived neurotrophic factor (BDNF), and neurotrophin 3 (Rolls et al., 2009). Thus, astrocyte migration to the injury site is crucial for perilesional neurons to survive (Liu and Chopp, 2016). The glial scar fills the ECM gaps in the lesion area, providing an environment where the vascularization network can regrow. Astrocytes and matrix components stimulate the local angiogenesis by recruiting endothelial cells and fibroblasts into the lesioned area (Rolls et al., 2009). Reactive astrocyte conditional ablation in transgenic mice leads to increased local tissue disruption, severe demyelination, and neuron and oligodendrocyte death (Bush et al., 1999; Faulkner et al., 2004; Vercelli and Boido, 2015), indicating that the glial scar might have an essential role in the injury acute phase. However, once a certain homeostasis level is reached, the glial scar impedes axon growth, necessary for repair. In that manner, the glial scar possesses a dual role, and its manipulation has to be well planned since its beneficial or detrimental role appears to be a matter of timing (Rolls et al., 2009).

### The Glial Scar and Brain Repair: Effects on Plasticity and Neurogenesis

Brain plasticity refers to any process that leads to the recreation of functional neuronal circuits and function regain. Plasticity involves short-distance axon sprouting, leading to new connections and alteration in the strength of existing connections (Sharma et al., 2013). These changes can allow signals to bypass areas of damage through newly created circuits and reassign areas of the CNS to new functions. After brain injuries, such as a stroke, neurons in the perilesional area upregulate signaling pathways that promote axonal growth and synapse formation (Dancause and Nudo, 2011). There is an enhancement of dendritic spine turnover, providing a substrate for new connections. Neurons in perilesional tissue can project new axonal by several millimeters into nearby cortical areas where new functional synaptic connections are formed (Nagappan et al., 2020).

The glial scar tissue is well known for its inhibitory effect on axonal growth. One of the main studied glial scar growth-inhibitory components is CSPG. CSPG has been shown to induce neurite retraction and growth cone collapse *in vitro* (McKeon et al., 1991). Also, *in vitro* studies comparing astrocytic cell lines revealed decreased axonal growth when astrocytes produced more CSPG (Fidler et al., 1999). Degradation of CSPG by chondroitinase AC allowed for axon growth at the lesion site, although there was increased local astrocyte activation (Coulson-Thomas et al., 2008).

Other brain ECM components, many of them present at the glial scar, can inhibit neuroregeneration, mainly by inhibiting axonal growth, remyelination, and plasticity, summarized in Table 1.

**TABLE 1:** Proteins in the CNS extracellular matrix that contribute to the inhibition of neuroregeneration after injury.

**Inhibitory protein**	**Function**	**Complementary receptors**
Nogo-A	Remyelination inhibitor via the RhoA pathway	Nogo-66 terminus: NgR1, p75, TROY, and LINGO1 Amino-Nogo terminus: unknown
MAG	Remyelination inhibitor via the RhoA pathway	NgR2, GT1b, NgR1, p75, TROY, and LINGO1
OMgp	Remyelination inhibitor via the RhoA pathway	NgR1
Versican (CSPG2)	Important during inflammation as it interacts with inflammatory leukocytes and inflammatory cells recruiting chemokines. It also stabilizes perineuronal nets to stabilize synaptic connections.	N-terminus: hyaluronan in the extracellular matrix (ECM) C-terminus: Ligands in ECM, especially tenascin
NI-35	Non-permissive growth factor in myelin	Unknown
Ephrin B3	Inhibits remyelination	EphA4
Semaphorin 4D (Sema 4D)	Inhibits remyelination	PlexinB1
Semaphorin 3A (Sema 3A)	In scars in both PNS and CNS injuries	Nrp1, Nrp2, L1cam, Nrcam

**Nogo*: Neurite outgrowth inhibitor; *NgR1*: Neuronal Nogo-66 receptor 1; *LINGO1*: Leucine rich repeat and Immunoglobulin-like domain-containing protein 1; *p75*: neurotrophin receptor; *TROY*: Tumor necrosis factor receptor superfamily member 19; *RhoA*: Ras homolog family member A; MAG: Myelin-associated glycoprotein; GT1b: Trisialoganglioside protein; OMgp: Oligodendrocyte-myelin glycoprotein; CSPG2: chondroitin sulfate proteoglycan core protein 2 or versican; ECM: extracellular matrix; NI-35 A: CNS myelin-associated neurite growth inhibitor; EphA4: Ephrin type-A receptor 4; Nrp1: Neuropilin 1; Nrp2: Neuropilin 2; L1cam: L1 cell adhesion molecule; Nrcam: Neuronal cell adhesion molecule. Extracted from Nagappan et al. (2020). The original material is available under the terms of the Creative Commons CC BY license. To view a copy of this licence, visit http://creativecommons.org/licenses/by/4.0/.*

The adult mammalian brain has two main areas known to produce new neurons: the subgranular zone (SGZ) of the hippocampus dentate gyrus, in which newborn neurons migrate laterally and integrate the hippocampus’s granular zone, and the subventricular (SVZ) located in both lateral ventricles. Neural stem cells (NSC) located in the SVZ are pluripotent stem cells (iPSC) that can differentiate into astrocytes, oligodendrocytes, and neurons. Newborn neurons (neuroblasts) from the SVZ migrate a long distance through the rostral migratory stream (RMS) to the olfactory bulbs, where they differentiate into mature interneurons (Alvarez-Buylla and Garcia-Verdugo, 2002). The discovery of adult neurogenesis in mammalian brains shed light on new possibilities for brain repair. In rodent models of brain trauma and stroke, there is increased cell proliferation in the SVZ and recruitment of neuroblasts that migrate along blood vessels toward the injury (Saha et al., 2013; Liang et al., 2019). Reactive astrocytes are essential players in this process. Astrocytes are critical regulators of adult neurogenesis. Astrocytes are one of the primary sources of molecules such as bone morphogenetic protein (BMP) and WNT, which regulate NSC proliferation and differentiation. NSC are attracted to the injury site by chemoattractive agents like CCL2 (C-C motif ligand 2), CCL11 (C-C motif ligand 11), CXCL12 (C-X-C motif ligand 12), and Prokineticin 2 (PROK2), mostly produced by astrocytes (Yan et al., 2007; Filippo et al., 2013; Moon et al., 2013; Mao et al., 2016; Wang et al., 2017; Zamproni et al., 2017; Mundim et al., 2019).

The glial scar matrix components also influence NSC cells under both physiological and pathological conditions. The developing CNS is enriched in proteoglycans which control developmental processes: neuronal migration and homing. In the adult brain, CSPG contributes to the maintenance of the neurogenic niches. Sulfated proteoglycan structures and, especially CSPG, were reported to affect NSC fate, survival, and maturation (Rolls et al., 2009). In a mouse model of brain trauma, SVZ-derived neuroblasts migrate toward the injured cortex but do not enter the area corresponding to the glial scar. Galindo et al. (2018) showed that, *in vivo*, neuroblasts migrated around the glial scar and attributed the inhibition of penetration into the scar to the presence of CSPG. *In vitro*, CSPG impaired neuroblast migration by altering cell protrusion and adhesion dynamics through Rho GTPase inhibition.

Although mobilization of NSC toward the injury occurs, many cells die or stray from the migratory path. Many NSC that reach the injury area fail to integrate into new neuronal circuits and die. For this reason, adult endogenous neurogenesis is insufficient for complete brain repair (Lu et al., 2017).

## Why Use Bioscaffolds to Repair the Brain?

To answer this question, we must first address the concept of repair. The term “repair,” when used to describe damaged tissue healing, means to restore tissue architecture and function and comprises two processes: regeneration and replacement. Regeneration occurs when the damaged tissue grows into new tissue and is restored to its normal state. Replacement occurs when a different tissue, usually connective tissue, is deposited over the damaged tissue, producing a scar (Krafts, 2010). The CNS anatomy, physiology, and pathobiology complexity make repair exceptionally challenging. Rebuilding the brain means rebuilding the complex brain tissue architecture and its intricate and extensive vascular networks, not only morphologically but also functionally (Xu et al., 2011). The disability provoked by cerebral lesions justifies the need to explore new therapeutic solutions (Nih, 2020).

As with most tissues in the body, the brain has mechanisms to regenerate itself, such as, previously mentioned, endogenous neurogenesis and neuroplasticity (Sharma et al., 2013). However, these processes are limited after injury (Modo, 2019). One of the main reasons explaining the limitation is the hostile microenvironment formed in brain injuries or diseases. The lack of a healthy ECM and the presence of the glial scar impairs neuronal survival, axonal sprouting, and synaptogenesis (Erning and Segura, 2020).

Tissue engineering is a newly emerging field that combines biomaterials, stem cells, and chemical and physical cues to produce engineered tissue-like structures with the ultimate goal of replacing *in vivo* tissues and organs (Chandra et al., 2020). Biomaterials refer to a class of materials that have been engineered to integrate with a biological system and provide beneficial effects by directing or controlling cell interaction (Detsch et al., 2018). In brain injuries, biomaterials are mainly used for two purposes: as bioscaffolds, to provide mechanical support to the injured brain while providing cues for new neural circuits formation, or as carriers, to deliver content such as stem cells, growth factors, exosomes, and gene vectors to the site of injury (Tuladhar et al., 2018). By replacing the virtual cavity formed after a brain injury, bioscaffolds can provide a tissue−appropriate physical and trophic environment for new neural cells and circuitry to survive and integrate into the host tissue (Tuladhar et al., 2018).

## Brain Ecm Composition Versus Biomaterial Characteristics

The ECM is fundamental for regulating several neural processes, including neurite outgrowth, synaptogenesis, synaptic stabilization, and injury-related plasticity, both in development and adulthood (Lam et al., 2019). Brain ECM is synthesized by both neurons and glia, comprising 20% of the adult brain’s total volume (Lam et al., 2019). The main components include glycosaminoglycans (chondroitin sulfate, heparan sulfate, and hyaluronic acid), proteoglycans (neurocan, brevican, versican, and aggrecan), glycoproteins (tenascin-R), and low levels of fibrous proteins (collagen, fibronectin, and vitronectin) (Lam et al., 2019). Also, brain ECM has a different composition in different compartments, such as the vascular basement membrane composed of collagen, laminin, fibronectin, and proteoglycans; the perineuronal matrix, made primarily of CSPG, and the interstitial matrix containing mainly proteoglycans, hyaluronic acid, and small amounts of collagen, elastin, laminin, and fibronectin (Lau et al., 2013). Due to the lack of fibrous proteins like collagen, the brain scar composition is softer than the healthy tissue (Moeendarbary et al., 2017).

The brain EMC structure imposes some characteristics for the biomaterial to be used in the brain. The material must be biocompatible and possess mechanical properties close to the brain tissue (stiffer materials lead to increased gliosis, softer materials lead to poor material stability at the implant site). The material should induce no or minimal inflammatory response. In this way, once long-term implants can cause a chronic inflammatory reaction, degradability is also desirable, and degradation products should be non-cytotoxic as well. Once the brain is confined to the skull, the biomaterial must present minimal swelling to avoid a rise in intracranial pressure (Tuladhar et al., 2018; Mitragotri and Lahann, 2009).

The presence of a rigid skull also influences the biomaterial delivery route, making injectable and shape-adaptable materials like hydrogels preferred over solid scaffolds that require invasive surgical procedures for the implant (Tuladhar et al., 2018).

## Biomaterials Are Used as Bioscaffolds in the Brain

Biomaterial scaffolds can be derived from both natural and synthetic materials (Chen et al., 2010). Natural materials include ECM proteins (collagen, fibrin, laminin), polysaccharides (alginate, chitosan), and decellularized tissue ECM. Synthetic materials include metals, ceramics, and inorganic polymers. Natural polymers are composed of naturally occurring biological substances and have properties closely resembling the native brain ECM. Natural materials possess bioactive molecules that can induce bioscaffold remodeling by the host, supporting *de novo* tissue formation and less prone to generating an immune response. However, its physicochemical properties are difficult to control (Modo, 2019; Tuladhar et al., 2018).

On the other hand, synthetic polymers are more tunable and can be more easily functionalized to achieve desirable characteristics (Tuladhar et al., 2018). Physicochemical properties and geometric conformation are precise, and they can be produced on an industrial scale. The absence of biological material reduces contamination risk but limits its ability to induce a regenerative response (Modo, 2019).

Biomaterials can assume different forms as particles, fibers and hydrogels (Tuladhar et al., 2018). Hydrogels are formed by physical or chemical cross-linking of hydrophilic polymers or by self-assembly systems. Their mechanical properties are usually similar to brain tissue. As previously mentioned, for brain repair, hydrogels are easier to deliver than solid scaffolds. They can be injected in liquid form, fill the irregular injury cavity and then polymerize, forming a gel (Lacalle-Aurioles et al., 2020).

## Bioscaffolds for Brain Repair

As we previously mentioned, bioscaffolds’ primary role is to provide a substrate where cells can anchor. The ideal bioscaffold should match the brain biochemical environment (water content and pH), the brain biophysical environment (viscoelastic properties and porosity), the ECM three-dimensional (3D) architecture on a biologically relevant length scale, and stimulate cell infiltration into it (Maclean et al., 2018).

Bioscaffolds’ mechanical forces can regulate the cell biological environment and control how cells interact with each other and with the ECM (Yuan et al., 2020).

**FIGURE 2:**
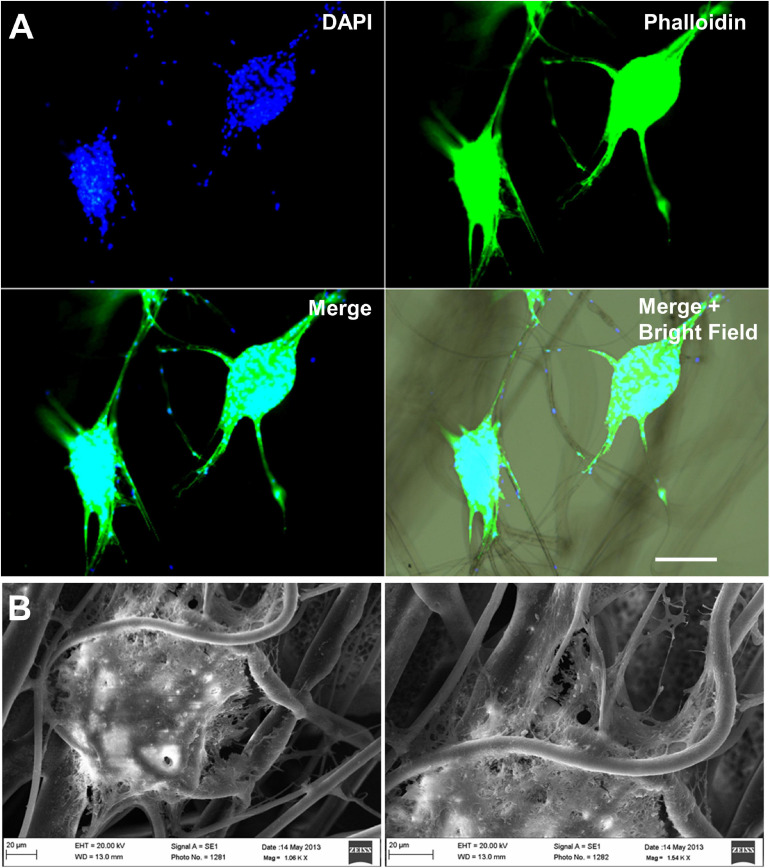
Fibrous bioscaffolds can direct neural stem cell migration. Mouse neural stem cells neutrospheres cultured over polylactic acid fibers migrate following the direction of the scaffold’s fibers. **(A)** DAPI plus phalloidin staining, Scale bar = 100 μm. **(B)** Scanning electron microscopy, Scale bar = 20 μm. Unpublished data from Zamproni et al. (2019).

Mechanical forces can influence cell functions such as migration, proliferation, differentiation, and apoptosis (Oksdath et al., 2018). For the CNS, bioscaffold electroconductive properties are usually desirable. It is well established that an electroconductive surface can increase neuronal differentiation, stimulate axon growing and facilitate synapsis formation (Herland et al., 2011; Pires et al., 2015). Fibrous scaffolds, particularly those with oriented fibers, can regulate and guide axon sprouting and synapse formation (Figure 2; Schaub et al., 2016; Zamproni et al., 2019). Since there is a tremendous variety in biomaterials and scaffold types being studied for their interaction with neural cells, Table 2 provides an overview of the most recent research on this field. Most studies involving biomaterials and neural cells are *in vitro*. There is a lack of *in vivo* data, which explains the absence of commercially available human therapy platforms so far. One of the concerns regarding implanting biomaterials in the brain is the foreign body response (Lotti et al., 2017; Mariani et al., 2019). Although some materials have been shown to modulate inflammation, for example, high molecular weight hyaluronic acid decreases microglia and glial scarring at the injury site (Austin et al., 2012), there is a concern of adverse immune reactions resulting in exacerbate inflammation, healing impairment, fibrotic encapsulation, and isolation and rejection of medical devices (Mariani et al., 2019). One of the strategies to overcome this issue is incorporating of bioactive molecules (cytokines or growth factors) that can modulate the inflammatory response. To date, bioscaffolds are being investigated together with stem cells, growth factors, and exosomes to increase therapeutic possibilities (Qu et al., 2020).

**TABLE 2:** Selected PubMed indexed papers published in 2020, focusing on bioscaffolds and neural cell interactions.

**Cell**	**Scaffold**	**Outcome**	**References**
Embryonic mouse cortical neural cells	Silk fibers	-Increased neurite extension -Guided axonal elongation -Guided cell migration from cellular spheroids along the fibers	Mercado et al., 2020
Rat hippocampal neurons	Graphene	-Induced neuronal networks formation -Increased GABAergic activity	Rauti et al., 2020
Mouse NG108-15 cells	Graphene oxide/silk fibers	-Increased cell proliferation -Increased neurite extension	Magaz et al., 2021
Neuroglioma cells	Poly (3,4-ethylenedioxythiophene)/chitosan fibers	-Increased cell proliferation -Increased axon density	Du et al., 2020
Mouse NG108-15 cells	Poly (3,4-ethylenedioxythiophene)-polystyrene sulfonate (PEDOT: PSS)/silk fibers	-Increased cell proliferation -Increased neurite extension	Magaz et al., 2020
Human neuroblastoma cells	Poly-(-caprolactone (PCL) nanofibers	-Increased neurite extension	Elnaggar et al., 2021
Human neuroblastoma cells	Poly(3,4-ethylenedioxythiophene) (PEDOT)/Carbon nanotubes	-Increased neuronal markers	Dominguez-Alfaro et al., 2020
Rat hippocampal cells	Aragonite skeleton of the coral **Trachyphyllia geoffroyi**	-Promoted elongation of astrocytic processes -Increased GFAP expression in astrocytes	Morad et al., 2020
Rat adipose tissue-derived neuron-like cells	Corning(§PuraMatrix(^TM^ hydrogel	-Increased cell proliferation -Increased neuronal markers expression	Darvishi et al., 2020
Rat pheochromocytoma cells	Porcine brain decellularized ECM	-Increased neuronal differentiation	Reginensi et al., 2020
Human Glioblastoma cells (U-87MG)	Carbon nanotubes	-Reduced cell growth	Parikh et al., 2020

## Bioscaffolds and Stem Cells

Stem cells are cells with self-renewal capacity and the potential to differentiate into different cell types (Zakrzewski et al., 2019). Stem cell-based therapies are hugely explored for the CNS. Stem cell sources include embryonic stem cells (ESC), mesenchymal stem cells (MSC), induced iPSC and NSC (Zakrzewski et al., 2019).

Stem cells can exert therapeutic properties by differentiating into appropriate cell types at the injury site or, more often, by secreting neurotrophic factors that can promote neuroprotection, angiogenesis, and neurogenesis (Zakrzewski et al., 2019).

As previously mentioned, the injured brain disrupted ECM does not offer a proper microenvironment for cell anchoring, proliferating, and differentiating (Li et al., 2016; Wan et al., 2015), and this poses a challenge for conventional stem cell delivery. MSC intravenous or intracardiac administration after a TBI in rats showed that <0.0005% of the cells injected were found at the injury site after 3 days (Turtzo et al., 2015). Delivering stem cells in bioscaffolds can surpass this issue once bioscaffolds provide the biomechanical support for cells until they can produce an ECM, increasing cell survival (Li et al., 2016). Scaffolds seeded with MSC improved cell retention compared to MSC alone in a mouse stroke model (Zamproni et al., 2019). Stem cells in biological scaffolds can be implanted into damaged sites to secrete neurotrophic factors, improve axon regeneration, promote myelinization, and reduce scar formation (Cooke et al., 2010). Table 3 provides a list of the most recent research focusing on stem cell delivery through bioscaffolds.

**TABLE 3:** Selected PubMed indexed papers published in 2020, focusing on bioscaffolds-stem cell delivery for CNS therapy.

**Stem cell source**	**Scaffold**	**Disease Model**	**Outcome**	**References**
Embryonic rat neural stem cells	Collagen/heparan sulfate porous scaffolds	Rat TBI model	-Improved regeneration of neurons, nerve fibers, synapses, and myelin sheaths -Reduced brain edema and cell apoptosis -Recovered rat motor and cognitive functions	Zhang et al., 2021
Human fetal brain- and spinal cord-derived neural stem cells	Aligned collagen sponge scaffolds	Rat complete spinal cord section	-Stem cell long-term cell survival -Stem cell neuronal differentiation -Reduced inflammation -Reduced glial scar formation -Recovered rat locomotor functions	Zou et al., 2020
Rat adipose tissue mesenchymal stem cells	RADA4GGSIKVAV (R-GSIK), a self-assembling nano peptide scaffold	Rat TBI model	-Reduced reactive astrocytes -Reduced microglial cells -Reduced TLR4, TNF, and IL6	Sahab Negah et al., 2020
Human umbilical cord mesenchymal stem cells	Collagen hydrogels	Rat Parkinson disease model	-No differences in proteomics between treated and control group	Santaella et al., 2020
Human umbilical cord mesenchymal stem cells	Collagen scaffolds	(1) Rat and dog complete spinal cord section (2) Patients with a medullar lesion	-Increased motor scores -Enhanced amplitude, and shortened latency of the motor evoked potential -Reduced injury area in magnetic resonance imaging	Deng et al., 2016
Adipose-derived mesenchymal stem cells overexpressing brain-derived neurotrophic factor (BDNF) and neurotrophin-3 (NT3)	Silk fibroin/chitosan scaffold	Rat complete spinal cord section	-Reduced scar tissue -Decreased inflammation -Increased nerve fiber formation	Ji et al., 2020
Rat neural stem cells	Matrigel	Rat complete spinal cord section	-Decreased reactive astrogliosis -Improved functional recovery	Wang J. et al., 2020
Human embryonic stem cell derived-neural stem cells	Hyaluronic acid hydrogel	Rat complete spinal cord section	-Increased oligodendrocyte differentiation -Improved locomotor function	Zarei-Kheirabadi et al., 2020
Mouse-induced pluripotent stem cell-derived neural stem cells	Fibroblast growth factor and chondroitin sulfate hydrogel	Mice stroke model	-Improved vascular remodeling -Improved cortical blood flow -Improved sensorimotor function	McCrary et al., 2020
Embryonic rat neural stem cells	Collagen/silk fibroin scaffold 3D bioprinted	Rat complete spinal cord section	-Reduced glial scar -Increased regenerative axons -Improved functional recovery -Improved electrophysiologic tests	Jiang et al., 2020

Moreover, bioscaffolds can direct stem cell fate by providing physical-chemical cues to enhance stem cell differentiation in one specific cellular type. Scaffolds’ physical cues include mechanical properties, pore sizes, porosity, surface stiffness, 3D structures, and mechanical and electrical stimulation. Scaffolds chemical cues include cell-adhesive ligands and exogenous growth factors (Xing et al., 2019). Table 4 summarizes the most recent research on bioscaffolds directing stem cell fate into neuronal types.

**TABLE 4:** Selected PubMed indexed papers focusing on bioscaffolds directing stem cell fate.

**Stem cell source**	**Scaffold**	**Outcome**	**References**
Mouse CGR8 embryonic stem cells	Poly ε-caprolactone (PCL)/gelatin scaffolds	-Promoted neural differentiation -Promoted efficient secretion of dopamine	Kheradmand et al., 2020
Human-induced pluripotent stem cell- and embryonic stem cell-derived neural stem cells	Poly(ethylene glycol) diacrylate-crosslinked porous scaffolds	-Increased neural cells functional maturity	Murphy et al., 2020
Human-induced pluripotent stem cells	Fibrin hydrogel	-Increased Olig2, MBP, Sox10, and PDGFRα expression -Increased oligodendrocyte differentiation	Nazari et al., 2020
Human olfactory ecto-mesenchymal stem cells	Chitosan-aniline pentamer/gelatin/agarose scaffolds	-Promoted differentiation into motor neuron-like cells	Bagher et al., 2019
Rat hippocampal neural stem cells	Poly-ε-caprolactone (PCL) fibers	-Increased cell proliferation -Increased astrocyte and oligodendrocyte differentiation	Patel et al., 2019
Neural stem cells	Poly (L-lysine) modified silk fibroin film	-Increased cell proliferation -Decreased apoptosis -Increased neuronal differentiation	Zhao et al., 2018
Mouse mesenchymal stem cells	Graphene foam	-Promoted dopaminergic neuronal differentiation	Tasnim et al., 2018

## Bioscaffolds for Growth Factor Delivery

Biomaterials are promising drug delivery vehicles for their ability to provide local, time-controlled release, which is particularly important in the brain since the BBB imposes intravenous drug delivery restrictions. Also, a biomaterial platform provides sustained drug release in a single application (Ziemba and Gilbert, 2017). Bioscaffolds can be used to deliver growth factors and help to create a pro-regenerative environment. Some of the factors include erythropoietin (EPO), BDNF, fibroblast growth factor (FGF), and vascular endothelial growth factor (VEGF) (Chan et al., 2017). Liu et al. (2019) combined a collagen/chitosan scaffold with FGF to promote recovery in a spinal cord injury model in rats. The authors found significant improvements in locomotor function and electrophysiological examinations 8 weeks after scaffold implantation. Rats receiving collagen/chitosan scaffold/FGF group presented improved nerve fibers tract regeneration in magnetic resonance imaging. Sandoval-Castellanos et al. (2020) developed a platform to increase neurite extension using heparin binding-functional amine groups. NGF and BDNF were bound to heparin by electrostatic interaction. Both NGF and BDNF, alone or combined, supported neurite growth. Maximum dorsal root ganglion neurite growth *in vitro* was found at 1 ng/mL NGF alone, without a BDNF addictive effect. Leipzig et al. (2010) compared interferon-gamma with BDNF and EPO surface-immobilized to a methacrylamide chitosan scaffold to promote rat NSC differentiation. Interferon-gamma was shown to be the best single growth factor for the induction of neuronal differentiation. Also, NSC exposed to interferon-gamma/chitosan scaffold resulted in more neurons than soluble interferon-gamma. Skop et al. (2016) engineered chitosan-based scaffolds by covalently linking heparin using genipin, which then served as a linker to immobilize FGF. Fetal rat NSC cultured over the FGF/chitosan scaffold proliferated and remained multipotent for at least 3 days without FGF addition to the medium. NSC seeded on this scaffold showed high expression of stem cell markers (BLBP and SOX2) and presented decrease GFAP astrocytic marker expression compared to cells maintained on fibronectin-coated plates with FGF supplemented media. These data suggest that FGF/chitosan scaffolds are efficient in maintaining NSC stemness.

## Bioscaffolds Combined With Exosomes

Exosomes are small membrane vesicles secreted by eukaryotic cells for intercellular communication and signaling (Ludwig and Giebel, 2012). Exosomes cargo includes cytokines and growth factors, signaling lipids, mRNAs, and microRNA that can influence cell response to injury, infection, and disease (Phinney and Pittenger, 2017). Exosomes have been studied for brain repair and now are being combined with bioscaffolds for therapy. This association rationale is that the scaffold may prolong exosome retention and sustain exosome delivery at the injury site (Tsintou et al., 2021).

Zhang et al. (2017) investigated if exosomes from MSC cultured in 3D collagen scaffold were superior for brain trauma recovery than exosomes from MSC cultured on conventional conditions. They delivered exosomes intravenously in a mice model of brain trauma. Both exosome types promoted endogenous angiogenesis and neurogenesis, reduced neuroinflammation, and significantly improved rat functional recovery. However, 3D cultured MSC-exosomes provided a better outcome in spatial learning than conventional MSC-exosomes.

Hsu et al. (2020) developed an alginate scaffold with human umbilical cord MSC exosomes to treat nerve injury-induced pain. The neuroprotective and neurotrophic effects of the exosomes were evaluated *in vitro*. The exosomes induced PC12 (pheochromocytoma) cells neurite outgrowth and protected PC12 and HEK293 (human embryonic kidney) cells against formaldehyde acid treatment. Right L5/6 spinal nerve ligation was performed in Sprague-Dawley rats to induce mechanical allodynia and thermal hyperalgesia. Exosomes in scaffolds were wrapped around ligated L5/6 spinal nerves for treatment. Treated rats performed better in functional scores and presented signs of enhanced myelinization of injured axons. Treatment also attenuated upregulation of c-Fos, GFAP, Iba1 (ionized calcium-binding adapter molecule 1), TNFα, and IL-1β, while enhancing IL-10 and GDNF (glial cell line-derived neurotrophic factor) in the ipsilateral dorsal root ganglion.

## Bioscaffolds and Gene Therapy

Gene therapy is fast-growing, and many CNS disorders are potential candidates for treatment approaches that involve the correction of genetic abnormalities (Choong et al., 2016). However, the use of viral vectors in gene therapy still poses some concern, and the development of new, highly efficient, low cytotoxic gene therapy strategies are required (Costard et al., 2020). Biomaterials and bioscaffolds may offer a safer alternative in delivering genetic material to cells and can be, in the future, the key for genic therapy in humans (Gower and Shea, 2013). Costard et al. (2020) used MgAl-NO_3_ layered double hydroxide as a non-viral vector to deliver nucleic acids (pDNA, miRNA, and siRNA) to MSC using a 3D scaffold approach. Nucleic acids were complexed with MgAl-NO_3_ layered double hydroxide and incorporated in collagen-nanohydroxyapatite scaffolds. The fabricated platform allowed successful MSC transfection.

The bioscaffold-gene therapy combination strategy has been investigated for the development of angiogenic platforms. Angiogenesis is a critical process required in the regeneration of many tissue and systems, including CNS regeneration (Lee et al., 2020). Laiva et al. (2018) developed a system by combining nanoparticles carrying a gene encoding for stromal-derived factor-1 alpha (SDF-1α) with a collagen-CSPG scaffold to enhance the MSC angiogenic response. They found that MSC on the scaffold exhibited early over-expression of SDF-1α mRNA combined with the activation of the angiogenic markers VEGF and CXCR4 (C-X-C chemokine receptor type 4). The conditioned media from these cells promoted a 20% increase in endothelial cell viability, a 33% increase in endothelial cell tubule formation, and a 50% increase in endothelial cell migration in a wound-healing model. Pro-angiogenic genes were also upregulated in endothelial cells exposed to conditioned media of MSC in scaffold.

Huntington’s Disease (HD) is an inherited autosomal-dominant neurodegenerative disease. Although genetic mutation responsible for HD is well know, there is still no treatment to stop or slow disease progression. Sava et al. (2020) developed chitosan nanoparticles loaded with anti-huntingtin siRNA to treat an HD mouse model using the intranasal route. The authors developed four formulations of nanocarriers able to lower huntingtin mRNA expression by at least 50%.

## Engineered Microtissue

### Microtissue Engineered Neural Networks

Microtissue engineered neural networks (micro-TENNs) were developed at the University of Pennsylvania for supporting neuronal survival and neurite extension (Struzyna et al., 2015). Micro-TENNs consist of neuronal populations with long axonal tracts entrapped into tubular hydrogels of 180–500 μm diameter and up to 2.0 cm length. Micro-TENNs are fabricated by filling a cylindrical mold with a longitudinally centered needle with liquid hydrogel. Once the gelification occurs, the needle is removed, creating a hollow micro-column that will be filled with an ECM solution. The ECM solution is responsible for providing an environment suitable for neuronal adhesion and axonal outgrowth (Struzyna et al., 2017). Micro-TENNs reconstitute the architecture of long-distance axonal tracts. They may serve as an effective substrate for re-establishing long-distance axonal connections and reconstruction of damaged brain pathways. Micro-TENNS have very small diameter being easily delivered into the brain with minimally invasive procedures.

Micro-TENNs were injected into rats’ brains using a stereotaxic device to connect deep thalamic structures with the cerebral cortex. The authors found that micro-TENN neurons survived at least 1 month and maintained their extended axonal architecture along the cortical-thalamic axis. They also found micro-TENN neurons extend neurites into the host cortex, with successful synapse formation (Struzyna et al., 2015). In another approach, Micro-TENNs were used to align an astrocytic network with mimicking the glial tube existent along the RMS. Those astrocytic networks successfully improved NSC migration and directly directed the cells from the neurogenic niche until the injured area (Winter et al., 2016).

### Biomaterial Based Cerebral Organoids

A cerebral organoid is an *in vitro* miniature organ resembling the brain. Organoids production relies on self-organizing cell properties, recapitulating early developmental events (Di Lullo and Kriegstein, 2017; Hoang and Ma, 2021). They are usually derived from iPSC and cultured for months with a set of growth and trophic factors that emulate organogenesis. The organoid organization is ideal for understanding cell interactions in a complex environment and offers great potential in disease modeling and regenerative medicine (Di Lullo and Kriegstein, 2017).

Bioscaffolds’ major role in tissue engineering is to control the biochemical and physical microenvironment of the cells. In organoids, cellular self-assembly leads to the secretion of ECM components and trophic factors by the cells themselves. However, it is desirable to control the initial conditions in organoid formation (Wan, 2016). In cerebral organoids, the most commonly used bioscaffold is Matrigel^®^. The generation of brain organoids based on Matrigel^®^ systems allowed to generate more sophisticated models that can capture region-specific features of the human brain, like cortical plate formation (Lancaster et al., 2017), forebrain (Kadoshima et al., 2013; Lancaster et al., 2017), midbrain and hypothalamic development (Jo et al., 2016; Qian et al., 2016). Matrigel^®^ droplets have been standardized for numerous brain organoid disease models such as microcephaly, AD, and PD (Hoang and Ma, 2021).

Lancaster et al. (2017) combined organoids with poly(lactide-co-glycolide) copolymer (PLGA) fiber microfilaments as a scaffold to elongate the embryoid bodies and found that the organoids engineered with microfilaments presented several advantages over the traditional organoid formation. The presence of PLGA microfilaments elongated and enhanced neuroectoderm formation and improved cortical development with microfilament-engineered cerebral organoids (enCORs) presenting large lobes of brain tissue. EnCORs showed a very reproducible neuronal induction and presented an almost complete lack of non-neural tissue with decreased amounts of endoderm and mesoderm layers. By reconstituting the basement membrane with Matrigel^®^, the authors could polarize the cortical plate and recapitulate an architecture similar to radial units, a characteristic not previously recapitulated *in vitro*.

### Three-Dimensional Bioprinting

As previously stated, the human brain is the most complex structure in the human body. In this context, 3D bioprinting offers a solution for designing specific individualized constructs while controlling tissue architecture. 3D bioprinting combines one or more cell types with a supportive bioscaffold, named bioink, to fabricate structures that resemble the native tissue topographically (Thomas and Willerth, 2017).

Despite the massive advance in the field in the last decade, it is still impossible to print whole tissues or organs that can be implanted. Some promising results have been shown for the spinal cord. Joung et al. (2018) printed a mixture of iPSC-derived spinal NSC and oligodendrocyte progenitor cells in a gelatin and fibrin bioink to fabricate a spinal cord. Bioprinted NSC was able to differentiate and extend axons throughout the scaffold. These neuronal networks’ activity was confirmed by physiological spontaneous calcium flux studies. Jiang et al. (2020) designed a 3D silk fibroin scaffold with cavities that simulate the normal spinal cord anatomy. They transplanted the scaffold combined with NSC in Sprague-Dawley rats submitted laminectomy. Rats receiving the combination of scaffold plus cells presented functional neurological scores significantly higher. They also performed better in electrophysiological studies, and magnetic resonance imaging revealed spinal cord continuity and injury cavity filling. The bioprinted spinal cords also decrease the glial scar while increasing regenerative axons.

Although implantable brain tissue is not yet available, it is feasible to produce smaller and less complex brain structures to study physiological cell-to-cell or cell-to-material interactions. 3D bioprinting can improve *in vitro* platforms for modeling neurological diseases, neural regeneration, and drug development. Li et al. (2020) developed a 3D brain-like co-culture construct where neurospheroid 3D structures were fabricated in an astrocyte-laden resembling a NSC niche environment. Then, the authors used a photo-cross-linkable bioink to bioprint neurospheroid layers. Neurospheroids into the 3D net were able to differentiate into neuronal cells. Sharma et al. (2020) used a fibrin-based bioink formulation combined with drug-releasing microspheres and human iPSC-derived NSC to print neural tissues. Microspheres were loaded with guggulsterone, a molecule capable of promoting NSC differentiation into dopaminergic neurons. Combining these three elements, they achieved a high viable tissue (95% viable cells 7 days post-printing) that expressed neural markers TuJ1 (class III beta-tubulin), Forkhead Box A2 (FOXA2), tyrosine hydroxylase (TH), GFAP, and the oligodendrocyte progenitor marker (O4). Quantitative polymerase chain reaction (qPCR) analysis also demonstrates the presence of *NURR1* (nuclear receptor related 1, gene expressed in midbrain dopaminergic neurons), *LMX1B* (LIM homeobox transcription factor 1-beta), *TH*, and *PAX6* (Paired box protein 6) after 30 days.

## Conclusion and Future Directions

Although spontaneous tissue regeneration is limited in the CNS, tissue engineering strategies to overcome the biological and physical challenges imposed by brain injury are gradually being developed.

Bioengineering already offers a series of commercially available products for tissues like skin, bone, and cartilage, but this is not the case for the CNS, and up to now, there are no suitable bioengineered therapeutic solutions to amend injuries to the CNS.

This review highlighted the CNS therapeutic approaches involving bioscaffolds. Since several pathologies can affect the CNS, it is rational to believe that these approaches will be complementary rather than competing, and the constructs should match patient needs. In PD, for example, a platform that stimulates dopaminergic neuron differentiation is required, whereas, in amyotrophic lateral sclerosis (ALS), the challenge is to replace long neuronal tracts. The advancement of precision medicine and new scaffold fabrication methods such as 3D printing will allow individualized treatment. Bioscaffolds and scaffold-based constructs should evolve in the next years with increasing complexity and functionality, impacting medical research.

However, significant challenges must be addressed. It is still impossible to produce fully vascularized tissue units, which is essential to increase constructs’ thickness and complexity while ensuring cell survival. Also, biomaterials’ long-term effects in the CNS and their interaction with the immune system must be addressed. Finally, it is essential to understand developmental biology to better design “smart bioscaffolds” capable of stimulating neurogenesis and neural network formation.

## Author Contributions

LZ and MM wrote the manuscript. MP revised and approved the manuscript. All authors contributed to the article and approved the submitted version.

## Conflict of Interest

The authors declare that the research was conducted in the absence of any commercial or financial relationships that could be construed as a potential conflict of interest.
